# Supplementary material for: In Silico Analysis and In Vitro Characterization of the Bioactive Profile of Three Novel Peptides Identified from 19 kDa α-Zein Sequences of Maize
Source: Molecules. 2020 Nov 19;25(22):5405. doi: 10.3390/molecules25225405 (PMC7699256; doi:10.3390/molecules25225405)
Supplement: Supplementary file 1 [file molecules-25-05405-s001.pdf]

molecules

MDPI

Supplementary material

In silico analysis and *in vitro* characterisation of the bioactive profile of three novel peptides identified from 19 kDa α-zein sequences of maize,

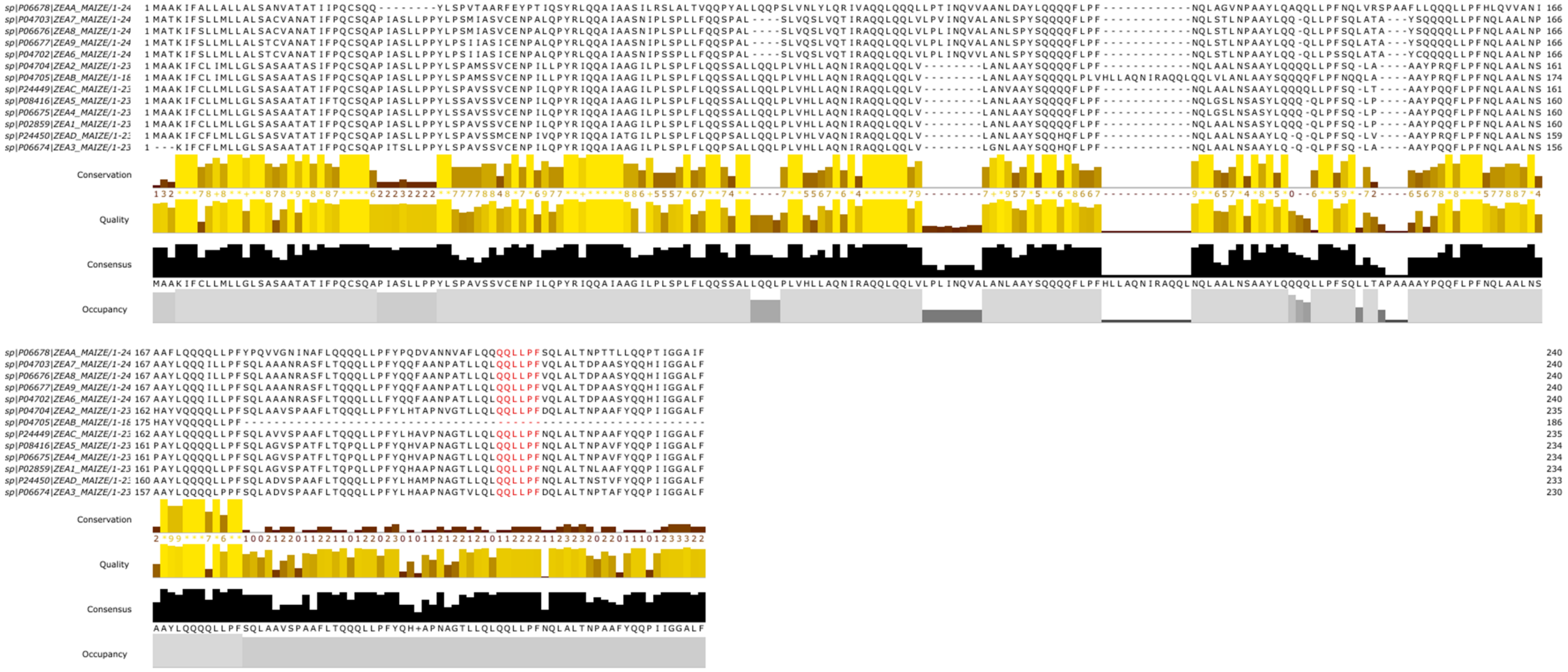

Figure S1. Sequence alignment of different 19 kDa α-zeins. Amino acids are expressed in one letter code format. Amino acids highlighted in red are the conserved sequenced motif of interest for the present study. Protein sequence alignment and conservation, quality, consensus and occupancy parameter calculations were performed using Jalview Software version 2.11.1.0. Reviewed 19 kDa α-zeins sequences were obtained from the Uniprot database (available at: https://www.uniprot.org/).

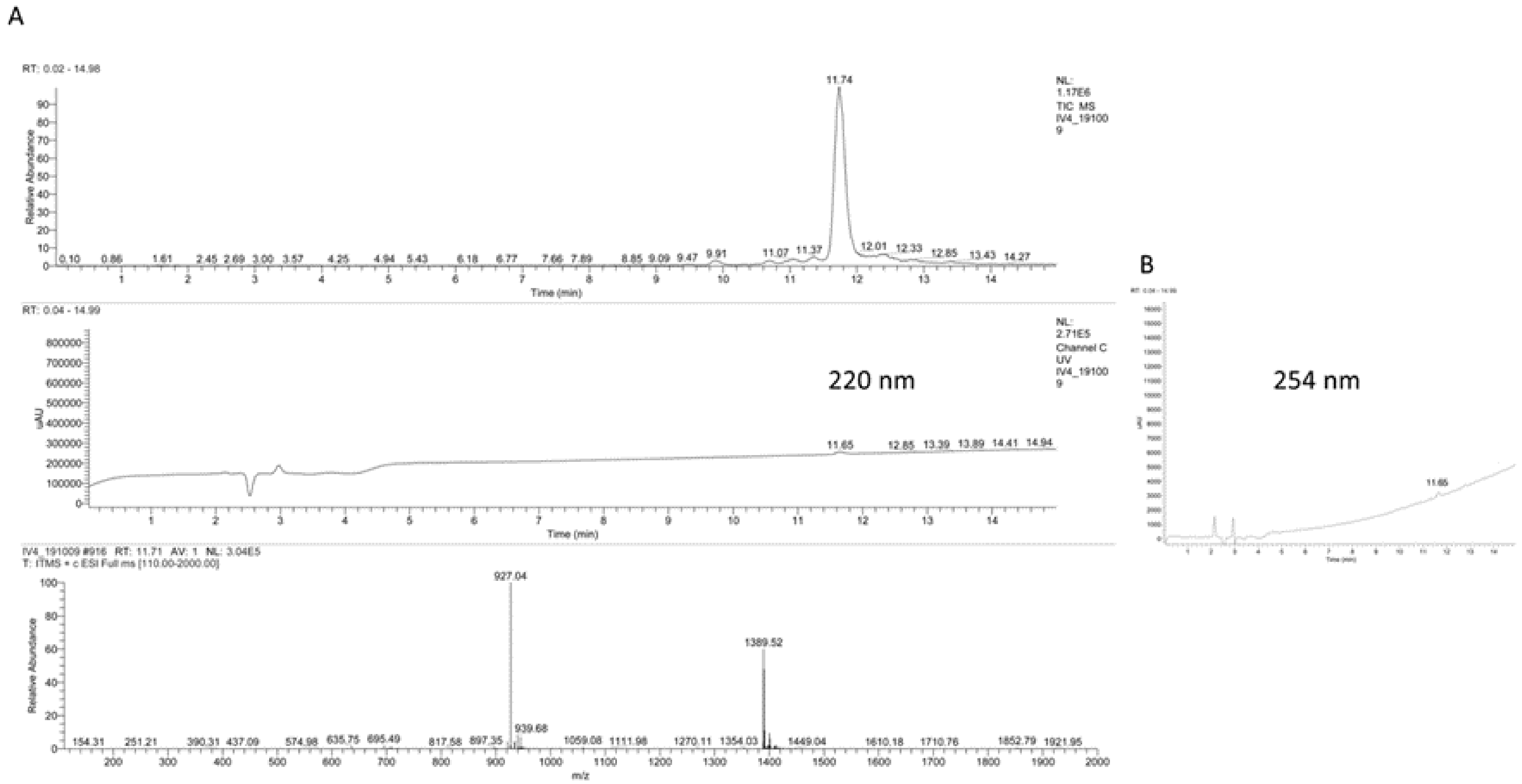

Figure S2. LC-ESI MS analytical data of 19ZP1 showing in (**A**) the ion current (upper), UV chromatogram at 220nm (middle) and full scan mass spectrum (lower), and (**B**) the UV chromatogram at 254 nm. The mobile phase consisted of a linear gradient of acetonitrile (20% to 70%) in 0.1% aqueous formic acid. *: Peak was further isolated by reverse phase high pressure liquid chromatography (RP-HPLC) with buffers 35%–50% B in A (A = 0.1% TFA in water; B = 0.1% TFA in acetonitrile).

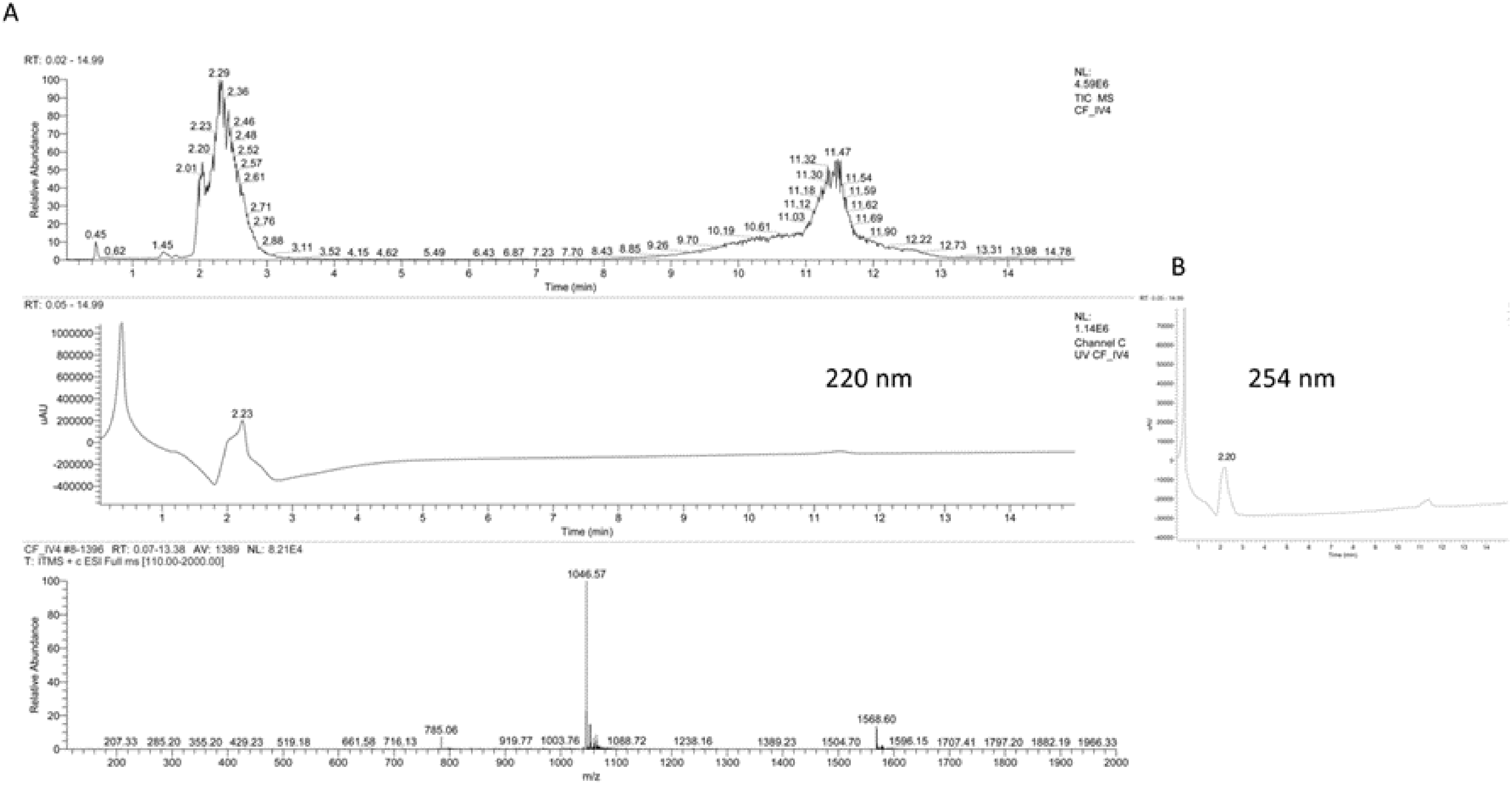

Figure S3. LC-ESI MS analytical data of CF19ZP1 showing in (**A**) the ion current (upper), UV chromatogram at 220nm (middle) and full scan mass spectrum (lower), and (**B**) the UV chromatogram at 254 nm. Chromatographic data of peptide samples used. Plot of analytical HPLC (left column) and ESI–MS spectrum (right column) of 19ZP1 (a), 19ZP2 (b) and 19ZP3 (c) peptides. The mobile phase consisted of a linear gradient of acetonitrile (20% to 70%) in 0.1% aqueous formic acid.

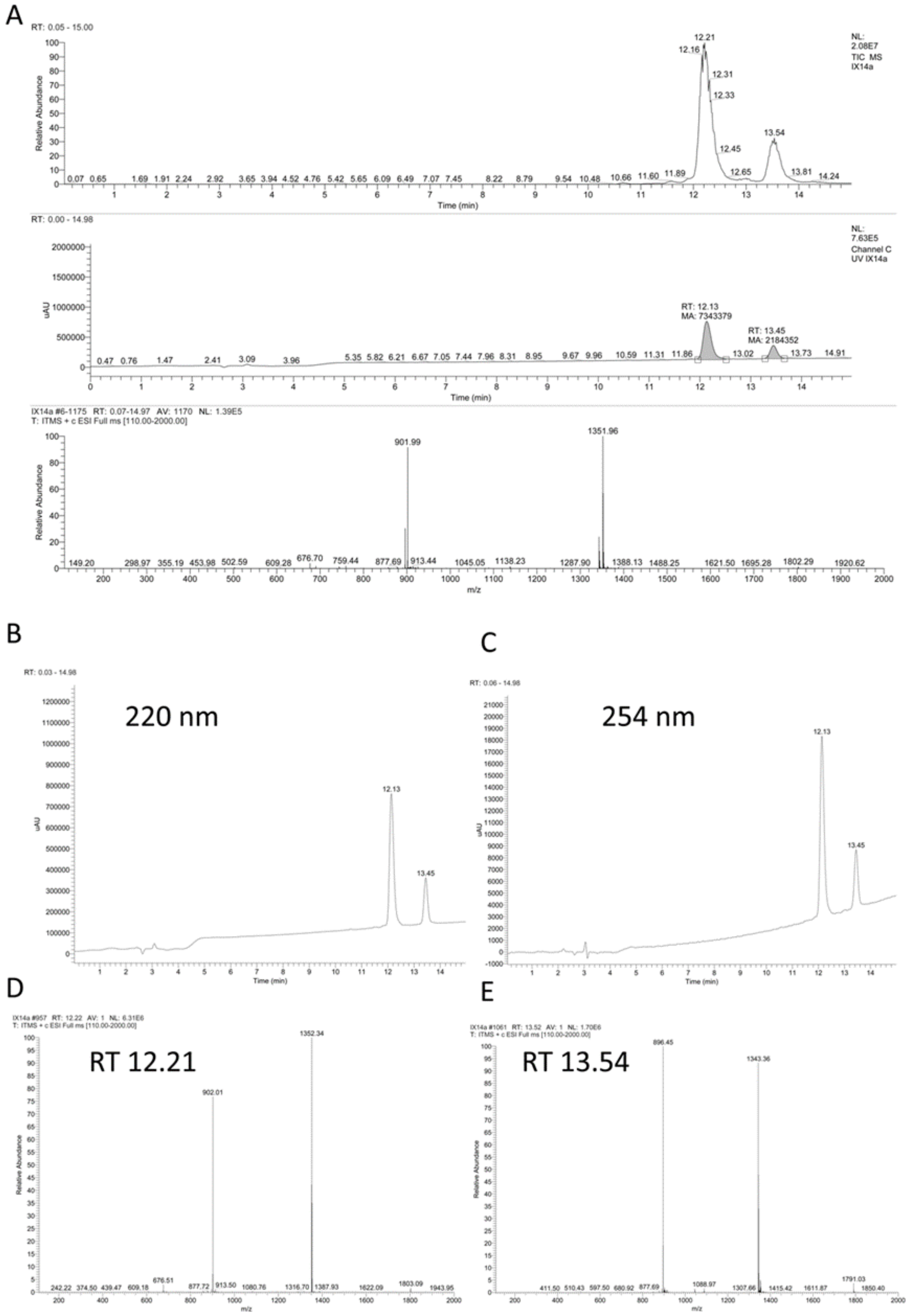
A
RT: 0.05 - 15.00
NL: 2.08E7 TIC MS IX14a
Relative Abundance
Time (min)
RT: 0.00 - 14.98
NL: 7.63E5 Channel C UV IX14a
uAU
RT: 12.13 MA: 7343379
RT: 13.45 MA: 2184352
IX14a #6-1175 RT: 0.07-14.97 AV: 1170 NL: 1.39E5
T: ITMS + c ESI Full ms [110.00-2000.00]
901.99
1351.96
m/z
B
220 nm
C
254 nm
D
RT 12.21
1352.34
902.01
E
RT 13.54
896.45
1343.36

Figure S4. LC-ESI MS analytical data of 19ZP2 showing in (**A**) the ion current (upper), UV chromatogram at 220nm (middle) and full scan mass spectrum (lower). Purity was calculated from the ratio of the two abundant peaks, where the peak at RT 12.13 is the product peak. UV chromatograms at 220 (**B**) and 254 nm (**C**). (**D**) ESI mass spectrum of the peak at RT 12.21, which corresponds to the product peak. (**E**) ESI mass spectrum of the peak at RT 13.54, which corresponds to a side product in which deamidation of the aspartate residue occurred. The mobile phase consisted of a linear gradient of acetonitrile (20% to 70%) in 0.1% aqueous formic acid. Peak at 12.3 min was further purified by reverse phase high pressure liquid chromatography (RP-HPLC) with buffers 35%–50% B in A (A = 0.1% TFA in water; B = 0.1% TFA in acetonitrile)

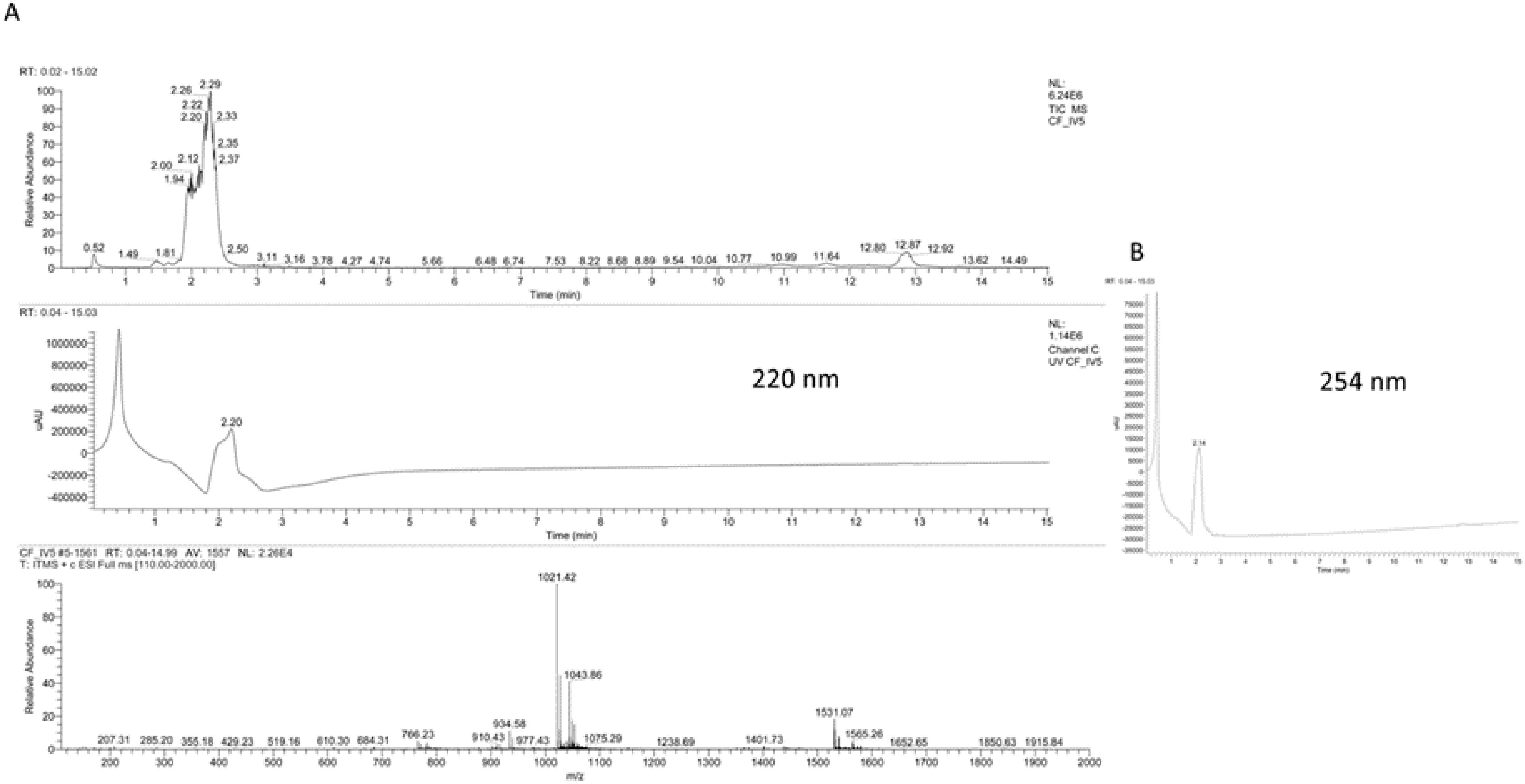

Figure S5. LC-ESI MS analytical data of CF19ZP2 showing in (**A**) the ion current (upper), UV chromatogram at 220nm (middle) and full scan mass spectrum (lower), and (**B**) the UV chromatogram at 254 nm. Beside the product, a side product (MW 3128 g/mol) is visible, which presumably represents a piperidide adduct of the product. The mobile phase consisted of a linear gradient of acetonitrile (20% to 70%) in 0.1% aqueous formic acid.

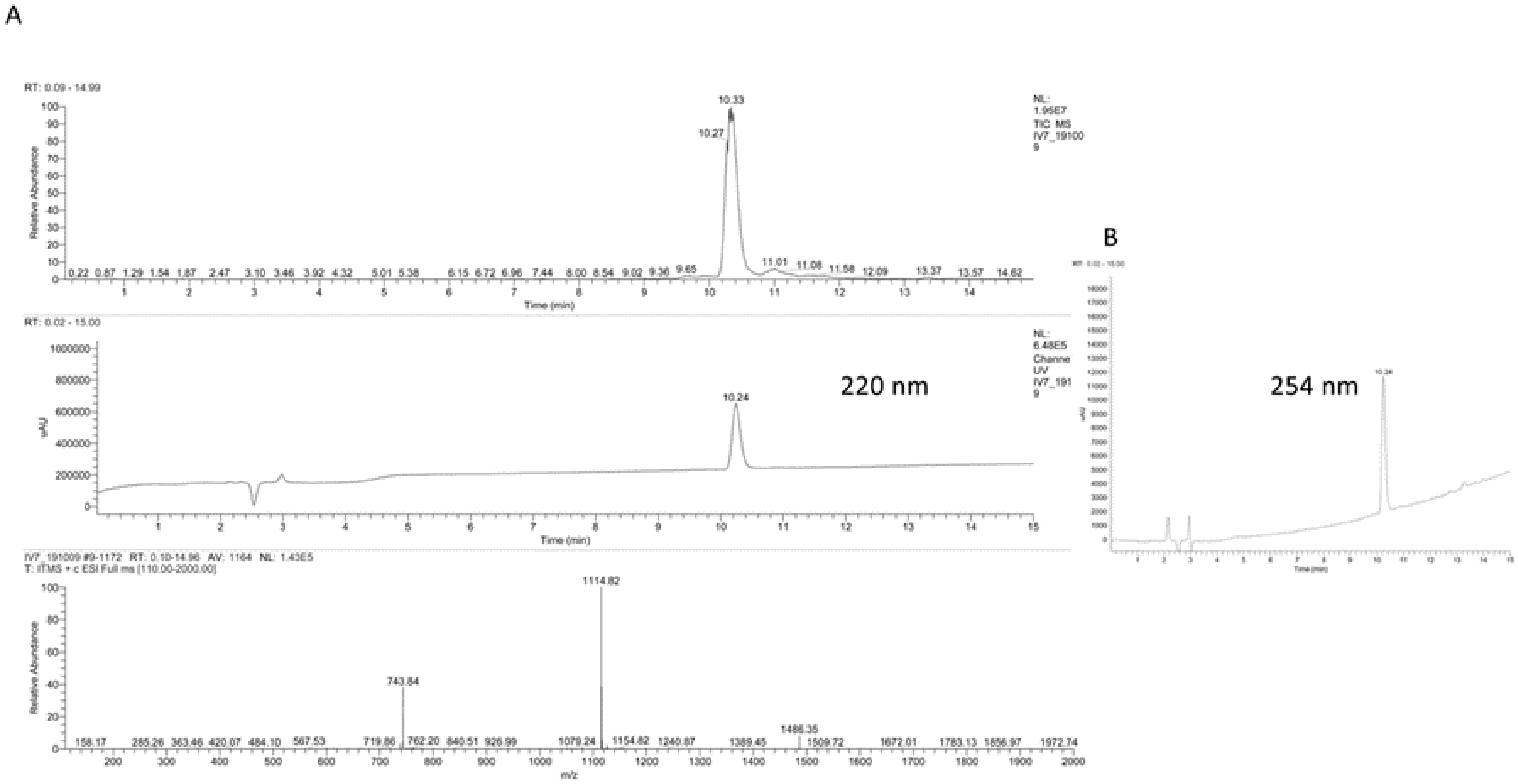

Figure S6. LC-ESI MS analytical data of 19ZP3 showing in (**A**) the ion current (upper), UV chromatogram at 220nm (middle) and full scan mass spectrum (lower), and (**B**) the UV chromatogram at 254 nm. The mobile phase consisted of a linear gradient of acetonitrile (20% to 70%) in 0.1% aqueous formic acid.

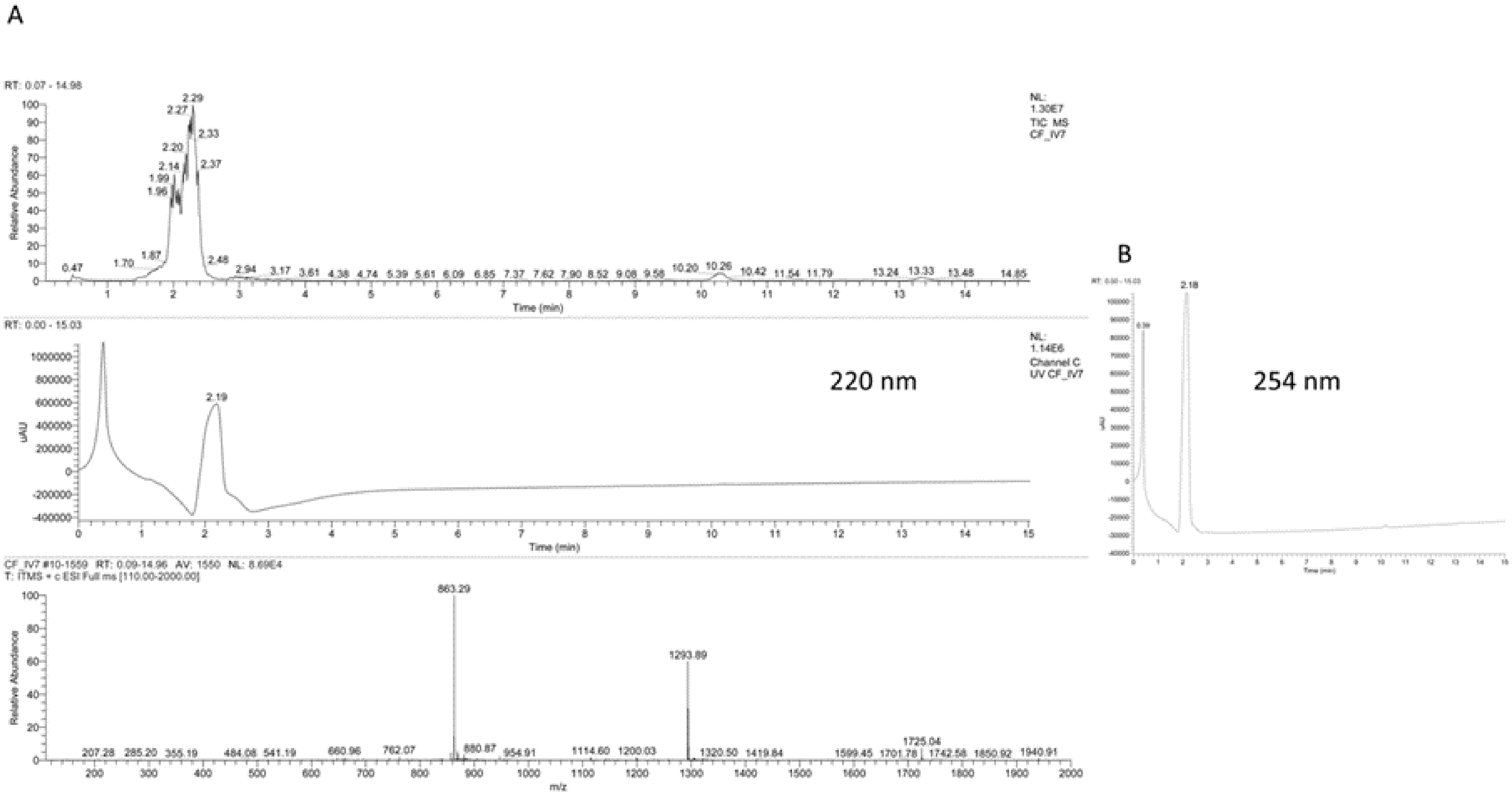

Figure S7. LC-ESI MS analytical data of CF19ZP3 showing in (**A**) the ion current (upper), UV chromatogram at 220nm (middle) and full scan mass spectrum (lower), and (**B**) the UV chromatogram at 254 nm. The mobile phase consisted of a linear gradient of acetonitrile (20% to 70%) in 0.1% aqueous formic acid.
